# Dual-network fiber-hydrogel membrane for osmotic energy harvesting

**DOI:** 10.3389/fchem.2024.1401854

**Published:** 2024-05-09

**Authors:** Licheng Cao, Huiqing Wu

**Affiliations:** College of Chemistry and Chemical Engineering, Donghua University, Shanghai, China

**Keywords:** dual-network, fiber-hydrogel membrane, osmotic energy harvesting, surface-space charge, synergistic effect

## Abstract

Osmotic energy harvesting was a promising way to alleviate energy crisis with reverse electrodialysis (RED) membrane-based technology. Charged hydrogel combined with other materials was an effective strategy to overcome problems, including restricted functional groups and complicated fabrication, but the effect of the respective charges of the two materials combined on the membrane properties has rarely been studied in depth. Herein, a new method was proposed that charged hydrogel was equipped with charged filter paper to form dual network fiber-hydrogel membrane for osmotic energy harvesting, which had excellent ion selectivity (beyond 0.9 under high concentration gradient), high ion transference number and energy conversion efficiency (beyond 32.5% under wide range concentration gradient), good property of osmotic energy conversion (∼4.84 W/m^2^ under 50-fold KCl and ∼6.75 W/m^2^ under simulated sea water and river water). Moreover, the power density was attributed to the surface-space charge synergistic effect from large amounts overlapping of electric double layer (EDL), so that the transmembrane ion transport was enhanced. It might be a valid mode to extensively develop the osmotic energy harvesting.

## 1 Introduction

In recent decades, the energy crisis has become a growing concern. Although the development of renewable energy sources, such as solar energy, wind energy, and tidal energy (Laucirica et al., 2021), has alleviated this problem to a certain extent, the collection and use of these clean energy sources are still limited by weather, geography, and time of the year. The osmotic energy, called “blue energy,” which has received extensive attention from researchers due to its advantages of large storage capacity, non-pollution and sustainability. It has been reported (Zhou and Jiang, 2020) that the theoretical total energy at the interface between seawater and river water can be up to 0.8 kWh m^−3^, and the global total energy can be up to 30 TW, which has a huge potential for acquisition. With the development of nanofluidics (Zhang Z. et al., 2021) and membrane science, reverse electrodialysis (RED) technology has become one of the effective means of osmotic energy harvesting, under which a variety of materials are used for the preparation of the corresponding membranes, such as two-dimensional materials (Zhang et al., 2019; Liu et al., 2020; Zhang L. et al., 2021), sulphonated polymers (Huang et al., 2019; Huang et al., 2020; Zhao et al., 2020; Sun et al., 2021), covalent organic frameworks (COFs) (Chen S. et al., 2021; Hou et al., 2021; Zuo et al., 2022), and polyionic liquids (Hu et al., 2022). Nevertheless, there are still some issues that limit their further application in osmotic energy harvesting, including complex preparation, high cost and high membrane resistance.

Compared with other materials, charged hydrogels have large amounts of three-dimensionally (3D) interconnected ion transport channels, enhancing the ion selectivity and transport. Currently, hydrogel membranes with different features and effects, including ample ion transport channels (Chen et al., 2020a; Chen et al., 2020b), enhanced ion interfacial transport efficiency (Zhang et al., 2020), and gradient structures (Chen Y. et al., 2021; Bian et al., 2021; Huang et al., 2023), have been reported for more effective osmotic energy harvesting and conversion. However, the complexity of the preparation of these hydrogel membranes, and the weak ion selectivity provided by limited functional groups still restrict their further applications. Therefore, selecting suitable materials to be combined with hydrogels for the preparation of corresponding membranes becomes a priority.

Cellulose-based materials are of interest due to their wide availability and lower cost. Presently, a number of cellulose materials, including wood (Chen et al., 2019; Chen G. et al., 2021; Chen J. et al., 2022), loofah fiber (Luan et al., 2022), bacterial cellulose (Wu et al., 2021; Sun et al., 2023) (BC), cellulose nanofibers (Liu et al., 2021) (CNF), etc., are often used to combine with charged hydrogels to prepare composite membranes. These strategies enhance ion conductivity, ion selectivity, mechanical and other properties, finally promoting the performance of osmotic energy harvesting. However, it is often unavoidable to use overly complicated treatment processes when using cellulosic materials (e.g., multiple alkali treatments at higher temperatures of naturally derived wood and loofah fibers to remove impurities, longer cycles of bacterial cultures to form cellulosic materials, etc.); besides, in the above research results, most of them investigated the effect of charge on the osmotic energy generation of one material without using the other, and little mention was made of the interaction between cellulosic materials and charged hydrogels (e.g., the link of charge effects). Compared to these cellulosic materials, low-cost filter papers tend to be more commercially available and their easy modification and hydrophilicity due to the abundance of hydroxyl groups have also been reported to be used in superhydrophilic-hydrophobic Janus-type interfaces (Kosak Soz et al., 2018), diagnostic systems (Böhm and Biesalski, 2017), and ultrasensitive strain sensors (Li et al., 2019), among others.

Herein, we report a charged fiber-hydrogel dual-network composite membrane combining charged filter paper and charged hydrogel obtained by modification of 2,2,6,6-tetramethylpiperidinium oxyradical (TEMPO) and used in osmotic energy harvesting. Due to the surface charge of N-filter paper and the space charge of hydrogel, the dual-network fiber-hydrogel membrane had excellent ion selectivity, high ion transference number and energy conversion efficiency (beyond 0.9% and 32.5% respectively under wide range concentration gradient), good current response stability and the property of osmotic energy conversion (∼4.84 W/m^2^). Moreover, it was further found that for the composite membranes corresponding to different charged filter papers, the composite membrane corresponding to the negatively charged filter paper had relatively better performance due to the enhanced ionic transport by the synergistic effect of surface-space charge. This provides a new strategy to improve the performance of osmotic energy conversion.

## 2 Experimental section

### 2.1 Materials and chemicals

2-acrylamido-2-methylpropane sulfonic acid (AMPS) was purchased from J&K Chemicals. Methyl methacrylate (MMA), acrylic acid (AA), 2,2,6,6-tetramethylpip-eridine oxygen radical (TEMPO), (3-Chloro-2-hydroxypropyl) trimethylammonium chloride solution (CHPTAC, 60 wt% aqueous solution) and lithium chloride (LiCl) were purchased from Aladdin. Methacrylic acid (MAA), [2-(methacryloxy)ethyl] dimethyl-(3-sulfonopro-pyl) ammonium hydroxide (SBMA) were purchased from Sigma-Aldrich.2-hydroxy-4’-(2-hydroxyethoxy)-2-methylphenylacetone (I2959), urea and Rhodamine 6G (R6G) were obtained from Adamas-beta. Sodium hydroxide (NaOH), sodium chloride (NaCl) and sodium bromide (NaBr) were obtained from Sinopharm. Co., Ltd. Sulfonated rhodamine (SR) was obtained from Shandong Yousuo Chemical Technology Co., Ltd. Sodium hypochlorite (NaClO, 10 wt% aqueous solution) was purchased from Ron reagent. Except for removing inhibitors, all the other chemicals are analytically pure and were used without any further purification.

### 2.2 Fabrication of negative-charged filter paper (N-filter paper)

The fabrication of N-filter paper was derived from Chen’s study (Xu et al., 2021). Briefly, 1 g filter paper was cut into pieces and then added into 100 mL aqueous solution containing 0.016 g TEMPO, 0.1 g NaBr and water at the speed lower than 100 rpm. After that, 3.722 g NaClO solution (10 wt%, 5 mmol/g filter paper) was dropwise added into the mixture. Then, 0.5 M NaOH was used to maintain the pH = 10.5 during the reaction for 2 h. The as-prepared N-filter paper was taken out, rinsed with plenty of water for three times to remove residual chemicals, dried in a 80°C oven for several minutes and soaked in 1 M HCl for 4 h. After soaking, the above washing and drying process was repeated again and finally obtained N-filter paper.

### 2.3 Fabrication of dual-network fiber-hydrogel membrane

Dual-network fiber-hydrogel membrane was fabricated by the following procedures. Firstly, the uniform precursor solution was prepared by adding AMPS (40 wt% in the solution), MMA, the third monomer (X, including MAA, AA or SBMA, the molar ratio of AMPS, MMA and X was certain), 1 mol% I2959 and 3 mol% MBAA (relative to the moles of monomers) into water and stirring for 30 min. It was noted that MMA and water should be firstly mixed to form emulsion, and when AMPS was added immediately and then the mixture was stirred violently, it would become clear after stirring. Then, the solution was added into a 0.1 mm-thick silicone mold containing N-filter paper. After putting it between two layers of glasses, removing the possible bubbles and irradiating with 365 nm UV light (the power was 50 W) for 90 min, the dual-network fiber-hydrogel membrane was obtained.

### 2.4 Characterizations and measurements

#### 2.4.1 Morphology characterization

The morphology of N-filter paper and dual network fiber-hydrogel membrane were confirmed by scanning electron microscope (SEM, Hitachi, SU8230).

#### 2.4.2 Fourier-transform infrared (FT-IR) spectra characterization

To obtain the information about hydrogen interaction or the functional group, the FT-IR spectra were carried out using Nicolet-iS50 spectrometer with ATR accessories.

#### 2.4.3 Zeta potential measurements

The Zeta potential was measured by using electrokinetic analyzer (Surpass3, Anton paar) with 1 mM KCl solution at different pH (ranging from 3 to 11).

#### 2.4.4 Ion selectivity measurements

The ion selectivity was determined by permeation experiment according to the previous study (Huang et al., 2019). In the experiment, R6G or SR solution (0.1 mM) was added to the left side of the H-type diffusion cell and deionized (DI) water was fed into the right side, respectively. The permeability was monitored using a spectrofluorophotometer (FLS1000, Edinburgh Instruments). According to the standard curve of R6G or SR, the permeable concentration was obtained.

#### 2.4.5 Relaxation time measurements

To record the relaxation time (T_2_) of the water molecular probe, VTMR20-010V-I low-field spectrometer (NIUMAG) was used. In the test, a Carr-Purcell-Meiboom-Gill (CPMG) sequence was used and the proton resonance frequency was 20.661 MHz. Besides, the π/2 and π pulse length in the CPMG sequence were 2.80 us and 4.64 us, respectively.

#### 2.4.6 Electrical measurements

The ion transport and osmotic power generation tests were performed using electrochemical workstation (CHI760E, Chenhua). The stability was tested by Autolab electrochemical workstation in I-t mode as the voltage ranging from −0.3 V to 0.3 V periodically (Each cycle maintained 10 min and the test lasted 6 cycles). To eliminate the redox potential automatically, a pair of commercial Ag/AgCl electrodes containing saturated KCl solution was used in the tests. Linear sweep voltammetry (LSV) was applied to obtain the I-V curve. According to the literature (Chen J. et al., 2022; Chen W. et al., 2022), the sweeping voltage ranged from −0.2 V to 0.2 V and the step voltage was 0.001 V. In this way, the open-circuit voltage (V_OC_) and short-circuit current (I_SC_) were directly acquired through recording the value of axis intercept. In the osmotic power generation test, two sides of the H-type diffusion cell were filled with high and low concentration solution, respectively. The testing membrane and PI membrane were mounted between two sides of cell, and the effective area of testing membrane was 0.03 mm^2^, the same as the previous works (Huang et al., 2019; Chen et al., 2020a; Chen et al., 2020b; Liu et al., 2021; Hu et al., 2022). By recording the current at the zero voltage and then calculating with the formula:

$$P=I^{2}R_{L}/S$$ (1)

The power density of testing membrane was obtained.

## 3 Results and discussion

### 3.1 Dual-network fiber-hydrogel membrane and its characterizations

**FIGURE 1:**
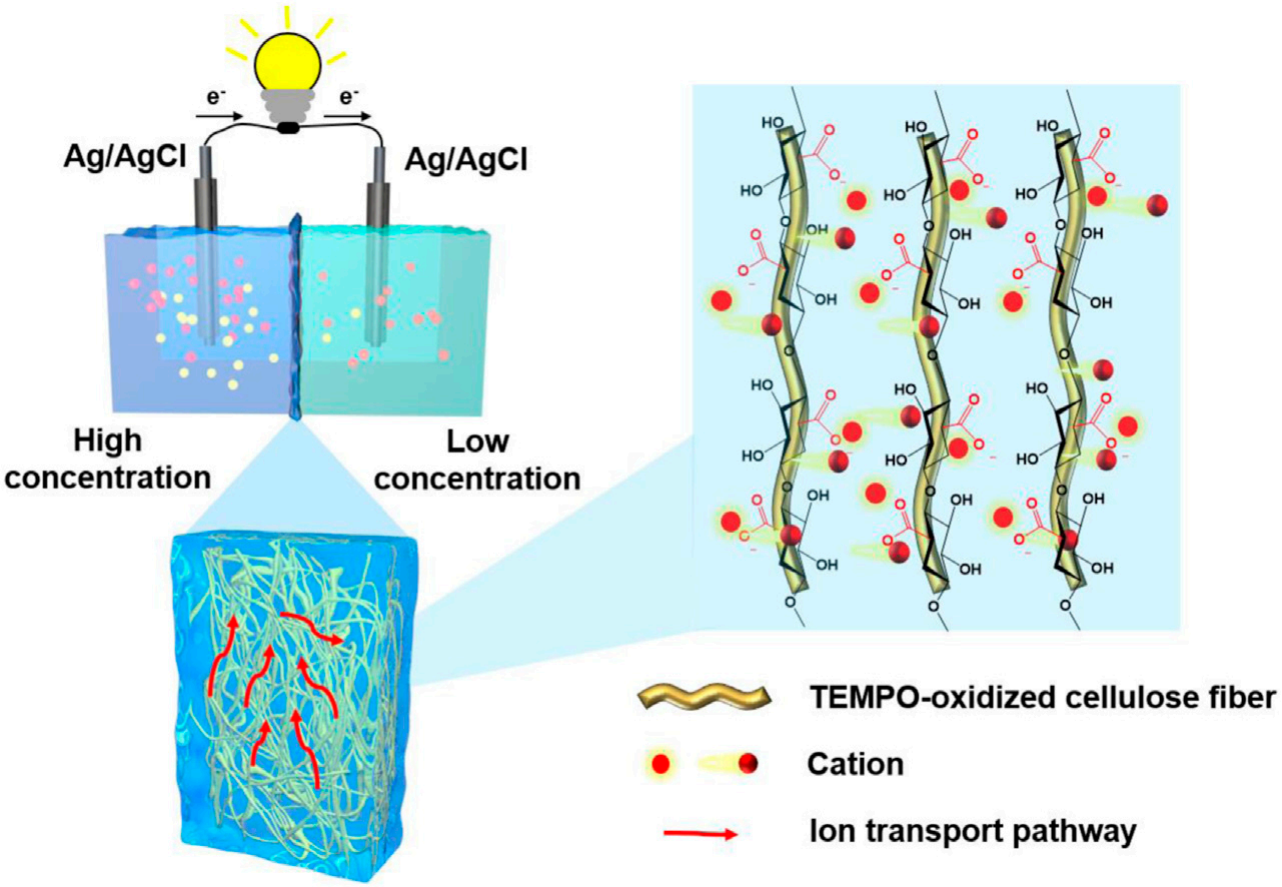
Schematic illustration of dual-network fiber-hydrogel membrane.

The schematic illustration is shown in Figure 1. Combining the N-filter paper fibers with the hydrogel matrix to form the structure of a dual network increases the number of paths for ion transport while increasing the cation transport flux. Based on it, the composite membrane has a higher ion mobility number and better ion selectivity than previously reported results, allowing more cations to migrate along a greater number of paths, increasing the corresponding current values and thus improving the performance of osmotic energy harvesting and conversion.

**FIGURE 2:**
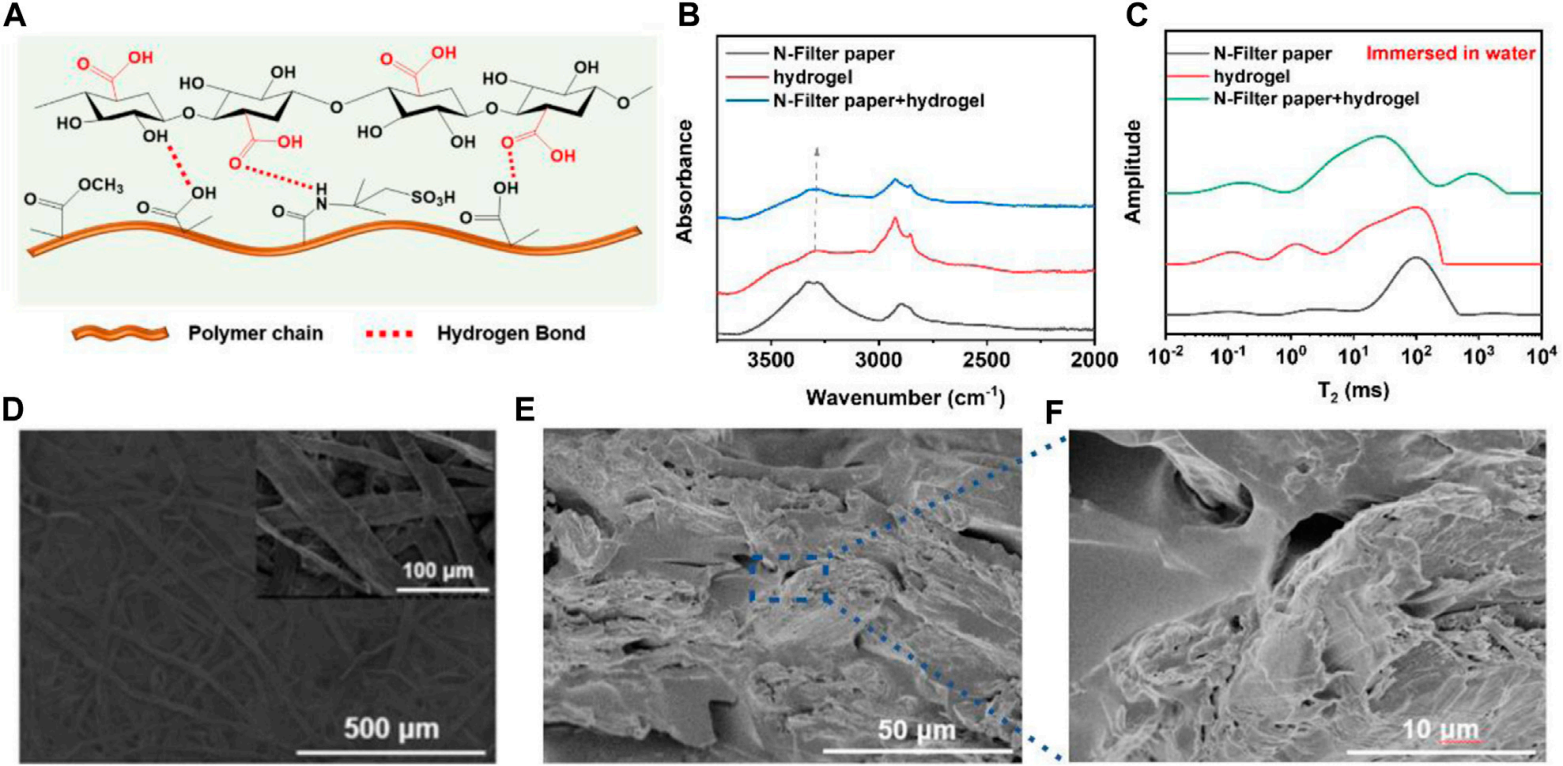
**(A)** The interaction of cellulose and polymer chain; **(B)** The peak shift of O-H in FT-IR image; **(C)** The T_2_ of H from water molecules of three kinds of samples had different states; **(D–F)** SEM images of N-filter paper and dual-network membrane.

For the interaction between the N-filter paper fibers and the hydrogel, which is shown schematically in Figure 2A, the interwoven gel polymer chain-charge-negative filter paper fiber dual network structure was formed by *in situ* polymerization through the infiltration of the hydrogel prepolymerization solution on the charge-negative filter paper fibers by taking advantage of the superhydrophilic nature of the filter paper. As shown in Figure 2B, the absorption peak at 3,297 cm^−1^ in the spectrum of hydrogel can be attributed to the stretching vibration of the O-H bond, whereas in the composite film sample the absorption peak is displaced to 3,289 cm^−1^, which indicates the existence of hydrogen bonding interactions between the charge-negative filter paper and the hydrogel (Zhang S. et al., 2021; Chen J. et al., 2022). In addition, characterising the interaction by means of low-field NMR measurements of the relaxation time of hydrogen in the water molecules, it can be seen that the peak of bound water with an initial relaxation time T_2_ < 50 ms is shifted to the left in the composite membranes, indicating that the water molecules in the membrane samples are more “tightly” bonded, as shown in Figure 2C. This “confinement” or binding of water molecules may be due to the interaction between the polymer molecular chains in the hydrogel and the cellulose molecular chains in the filter paper, which regulates the pore size of the composite membrane and verifies the formation of the dual network structure. The source of this restriction might be the interaction between cellulose molecular chains and polymers, thus adjusting the pore of membrane and proving the establishment of dual-network structure. SEM results of the N-filter paper (Figure 2D) and composite membrane samples also showed that the filter paper itself has a network structure with interlocking fibers, while the cross-sectional structural characterisation of the membranes in Figures 2E, F suggests that the hydrogel binds well to the filter paper fibers. Taken together, due to the interaction between the negatively charged filter paper fibers and the hydrogel as well as the introduction of the hydrogel matrix, the good combination of the two lays the foundation for the subsequent performance testing of the composite membrane, while the hydrogel fills up the larger pores in the filter paper and regulates the pore size of the membrane.

### 3.2 Ion transport properties of dual-network fiber-hydrogel membrane

**FIGURE 3:**
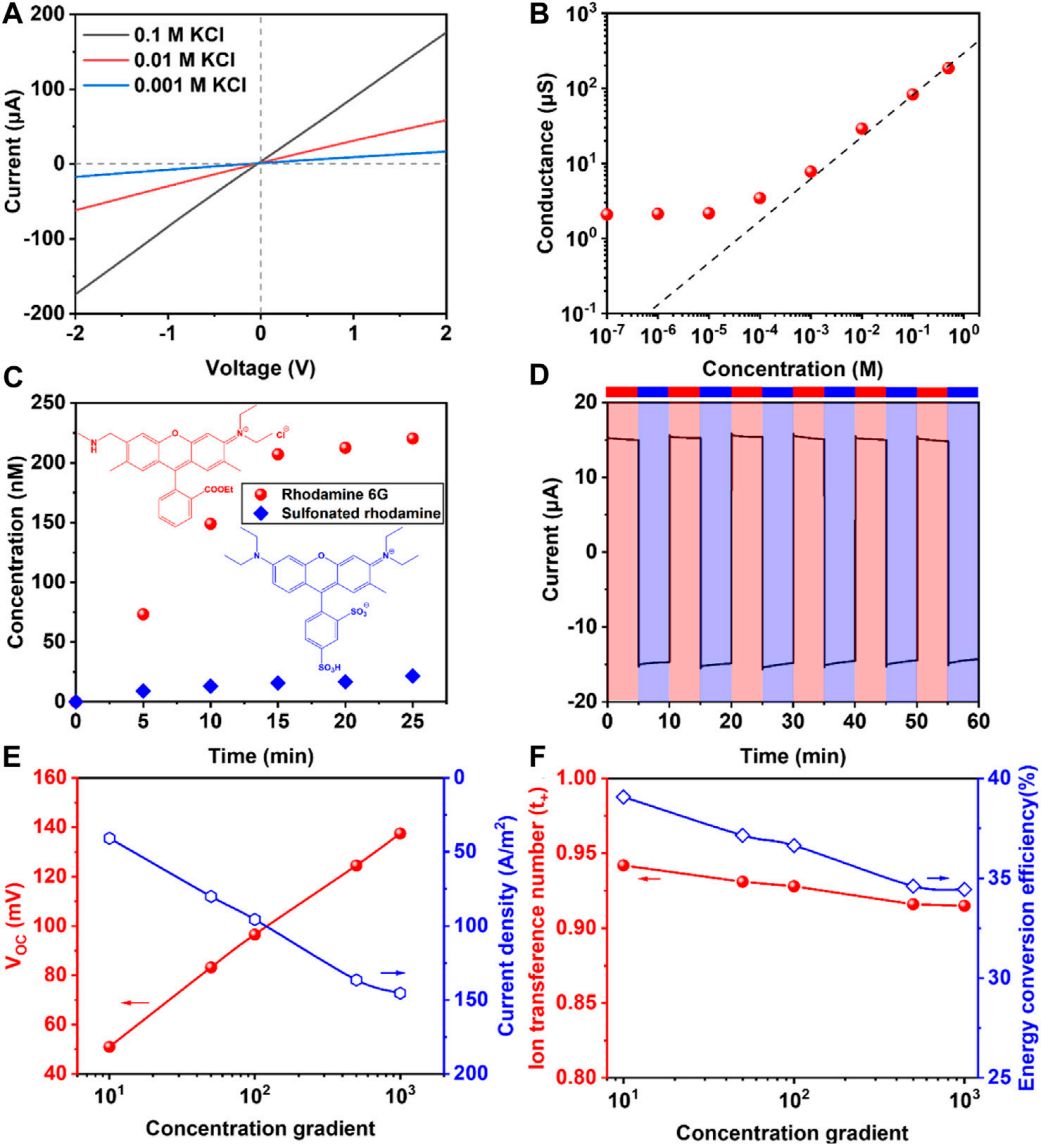
**(A)** The I-V curves of membrane at 0.1/0.01/0.001 M KCl; **(B)** The conductance of the membrane at different concentration; **(C)** Comparison of R6G and SR’s permeation rate within 25 min; **(D)** The I-t curve at the −0.3 V and +0.3 V to test the stability of membrane; **(E,F)** The changing of V_OC_, I_SC_, ion transference number and energy conversion efficiency at different concentration gradient (Low concentration was set as 1 mM).

To investigate the ion transport behavior of the composite membrane, the composite membrane was placed in the center of a symmetric H-type diffusion cell and the I-V curves were tested when the two reservoirs of were filled with the same concentration of KCl solutions. As shown in Figure 3A, the linear curves obeyed the Ohm’s law, indicating that the membrane had symmetrical structure. Subsequently, different ion transport behaviors were determined. In Figure 3B, the curve’s trend was almost linear when the concentration was higher than 10^−3^ M, while the conductance deviated obviously from the bulk curve when the concentration was below 10^−3^ M, which was attributed to the fact that the ion transport was dominated by the surface charge (Chen J. et al., 2022).

Ion selectivity was one of the most important properties for the membrane in osmotic energy harvesting. Therefore, in order to further explain the ion selectivity, two kinds of molecules with similar volume and opposite charge polarity, R6G and SR, were used in the experiment. In Figure 3C, the permeating concentration and permeating rate of R6G was much higher than that of SR, which proved excellent cationic ion selectivity. It could be attributed to the surface charge from N-filter paper and the space charge from hydrogel.

Besides, in order to evaluate the stability of membrane, the current test was taken with each cycle lasting 10 min in 0.1 M KCl solution, and the voltage alternated between +0.3 V and −0.3 V, respectively. In the I-t curve of membrane in Figure 3D, both positive and negative current still maintained at the same level after 60 min, showing the good stability. In Figure 3E, when the low concentration was set as 1 mM, the V_OC_ and I_SC_ increased with the growing concentration gradient. What’s more, in Figure 3F, we calculated the ion transference number (*t*_+_) and energy conversion efficiency (*η*). The calculating formula of them were as follows:

$$t_{+}=\frac{1}{2}\left[\frac{E_{diff}}{\frac{RT}{zF}\ln\left(\frac{c_{H}\alpha_{H}}{c_{L}\alpha_{L}}\right)}+1\right]$$

$$\eta =\frac{1}{2}{\left(2t_{+}-1\right)}^{2}\times 100\%$$

Here, *E*_diff_ was diffusion potential; *R*, *T*, *z*, *F* represented ideal gas constant, thermodynamic temperature, the valence of ions and Faraday constant, respectively. $c_{H}$ , $c_{L}$ , $\alpha_{H}$ , $\alpha_{L}$ represented high (low) concentration and their ionic activity coefficient.

The results in Figure 3F indicated that when the concentration gradient was large (1,000 fold), the *t*_+_ was beyond 0.9 and the energy conversion efficiency closed to 35%, showing great ion selectivity and efficiency, which was higher than results reported in other studies.

### 3.3 Dual-network fiber-hydrogel membrane for osmotic energy harvesting

Many factors affected the osmotic energy harvesting performance of dual-network fiber-hydrogel membrane. Firstly, in the direction of forward concentration gradient direction, when the molar ratio of AMPS to MMA was 1.5:1, the membrane resistance was relatively low (Supplementary Figure S2A). At the same time, the performance of osmotic energy harvesting was further evaluated via using a variable external resistance. Supplementary Figures S2B–D, S3 demonstrated the current densities and power densities generated by membrane-based power generators with different molar ratio and crosslinking agent’s mole fraction. According to the results of calculation (using Eq. 1), the maximum power density was relatively higher (3.7 W/m^2^) when the molar ratio was 1.5:1 and MBAA was 3 mol%. Besides, the power density of membrane could be adjusted by introducing the third monomer or replacing MMA with HEMA (Supplementary Figures S4–S7). These results showed that the molar ratio of AMPS, MMA, MAA was 1.5:1:0.9 satisfied the optimum condition.

**FIGURE 4:**
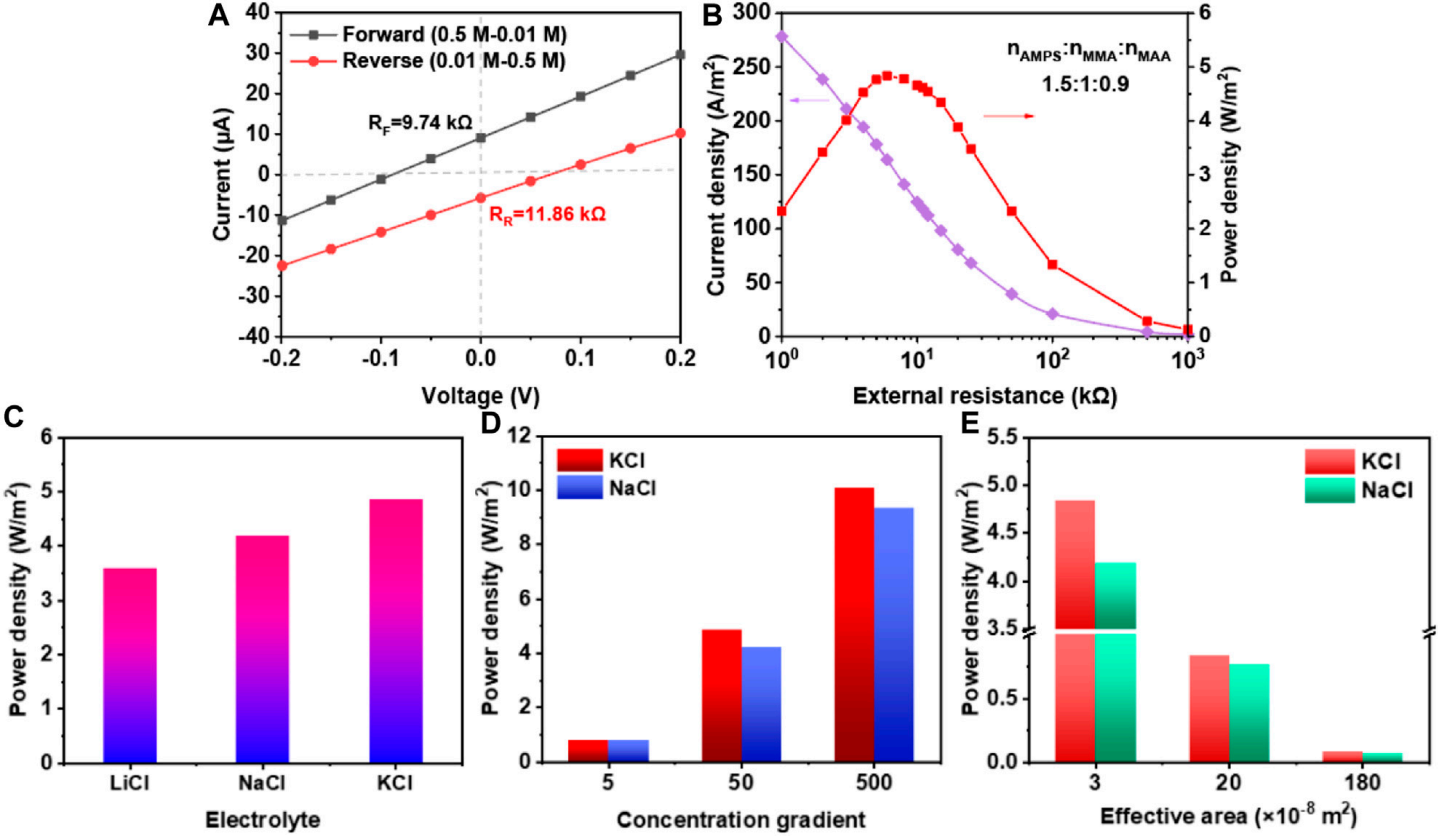
The osmotic energy conversion of membrane when molar ratio of AMPS, MMA, and MAA was 1.5:1:0.9. **(A)** The I-V curves of membrane at 50-fold concentration gradient with opposite directions; **(B)** The current density and power density curves at different external resistance; **(C)** Power density of different electrolyte; **(D,E)** Power density at different concentration gradient and effective area when using KCl and NaCl as electrolytes.

Figure 4A exhibited the I−V curves measured in 50-fold KCl gradient under forward and reverse diffusion directions. When the direction was forward, the V_OC_ and I_SC_ were −88.48 mV and 9.084 μA, respectively. Thus, the internal resistance of membrane was 9.74 kΩ. Nevertheless, it increased to 11.86 kΩ as the gradient direction was reversed. These phenomena resulted from the ion transport behavior, which included the diffusion and drift. Due to the same drift direction, under the reverse direction, the short-circuit current was relatively lower. In Figure 4B, in this condition, the current density decreased with the increasing external resistance. When the internal resistance of membrane was equal to the external resistance, the maximum power density was acquired (4.84 W/m^2^).

Furthermore, the power density was tested to investigate the membrane’s performance in different conditions. In Figure 4C, the power densities of 50-fold KCl, NaCl and LiCl were 4.84 W/m^2^, 4.19 W/m^2^ and 3.58 W/m^2^, respectively. The highest power density for KCl was ascribed to the lowest hydrate ionic radius and the largest diffusion coefficient. In Figures 4D, E, the osmotic energy conversion of membrane with different gradient and effective area were tested. The power density under 5-fold, 50-fold and 500-fold KCl were 0.812 W/m^2^, 4.84 W/m^2^ and 10.08 W/m^2^, respectively. At the same time, under 500-fold NaCl, the power density was a little lower (9.32 W/m^2^), which was caused by the larger hydrated ionic radius of Na^+^ than K^+^. Besides, as the effective area increased, the power density decreased. These results above proved that the membrane had good osmotic energy conversion properties.

**FIGURE 5:**
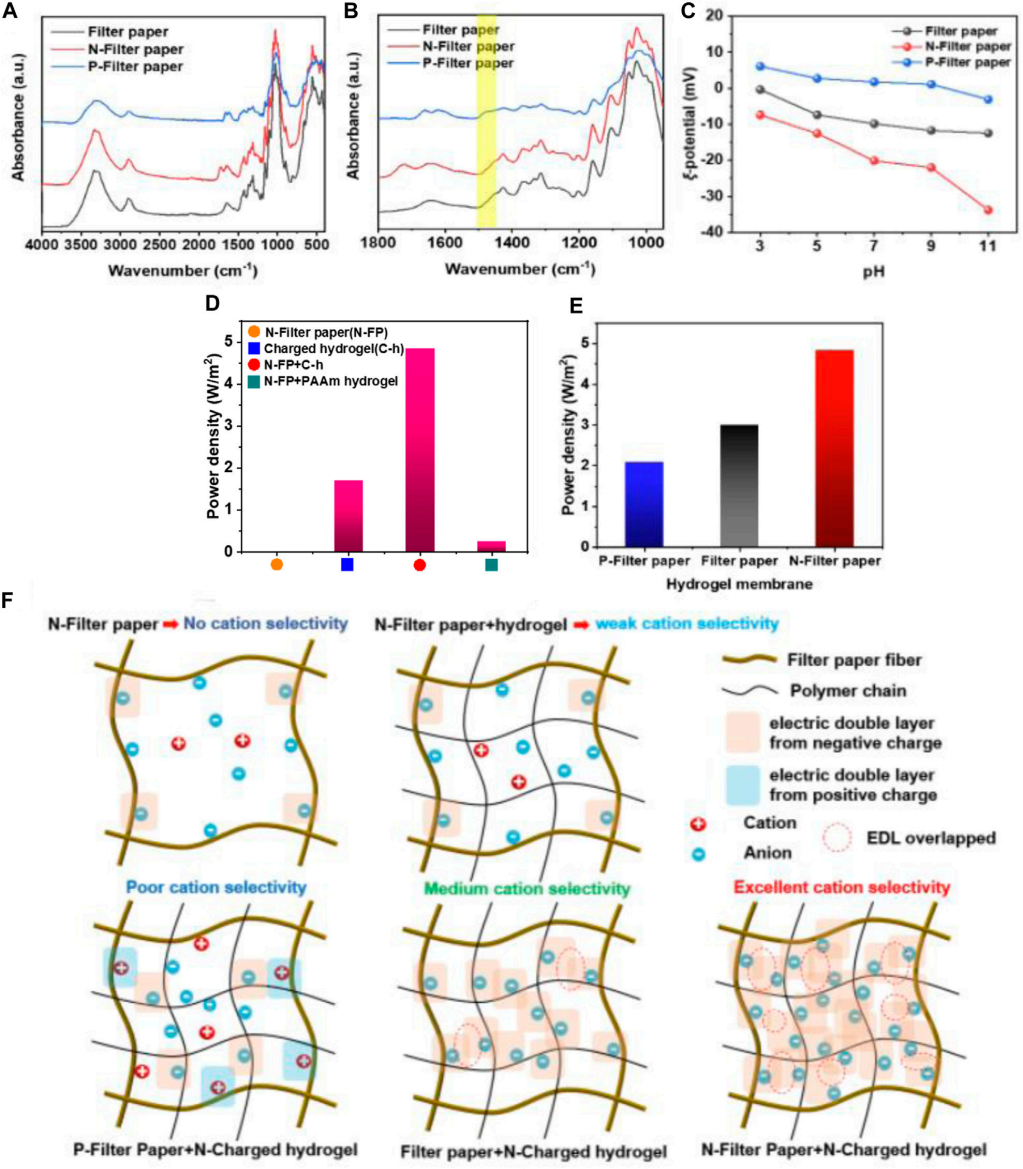
**(A,B)** The FT-IR characterization of different filter-paper samples; **(C)** The zeta-potential of different filter-paper samples; **(D)** Power density of four kinds of membranes; **(E)** Power density of membranes with different filter-paper samples; **(F)** The schematic illustration of the surface-space charge synergies model.

To further explore the relationship between the charge polarity of filter paper fiber and the osmotic power generation of membrane, the cellulose in the filter paper fibers was etherified and cationized by CHPTAC to make the filter paper fibers positively charged, and composite membranes were prepared in a similar manner. Firstly, in Figures 5A, B, the peak at 1,477 cm^−1^ in the spectrum of P-filter paper could be attributed to the vibration of the C-N bond, which can prove the success of the positively charged modification of the fibers. Then, in Figure 5C, the zeta potential of the N-filter paper and P-filter paper at pH = 7 were −20.1 mV and 1.77 mV, respectively. In Figure 5E, the power density of different charged fiber-hydrogel membrane had significant differences, and the N-filter paper-hydrogel membrane had the better power density, which meant the synergies of the surface charge from the N-filter paper and the space charge from hydrogel. In Figure 5D, the N-filter paper with micrometer-sized pores had no overlapping electric double layer (EDL), resulting in no power density, while hydrogel had far lower power density than dual network fiber-hydrogel membrane. At the same time, the hydrogel membrane without N-filter paper showed a relatively lower power density. Meanwhile, when the charged hydrogel in the fiber-hydrogel membrane was replaced with non-charged PAAm hydrogel, the power density was also far lower (only 0.24 W/m^2^). From the results above, it proved the necessity of combining negative fibers with charged hydrogel, which implied that the synergies of surface charge and space charge played a key role in improving osmotic energy harvesting. From the ion transport mechanism analysis, high ion selectivity mainly depends on the membrane pore size and chargeability, and the formation of the effect here is mainly because of the composite membrane in the filter paper fiber surface charge and hydrogel space charge formed a large number of bilayer overlap, triggering the synergistic effect of the two, which effectively improves the performance, and in the case of no hydrogel composite or only non-charged hydrogel composite, the bilayer overlap is little, no overlap or even the opposite charge is depleted and thus the performance is lower, which is shown schematically in Figure 5F. Based on the results and analyses above, it could be proved that the surface-space charge synergies affected the osmotic energy harvesting to large extent. It was worth noting that this result was firstly reported in the field of hydrogel based on other researches.

**FIGURE 6:**
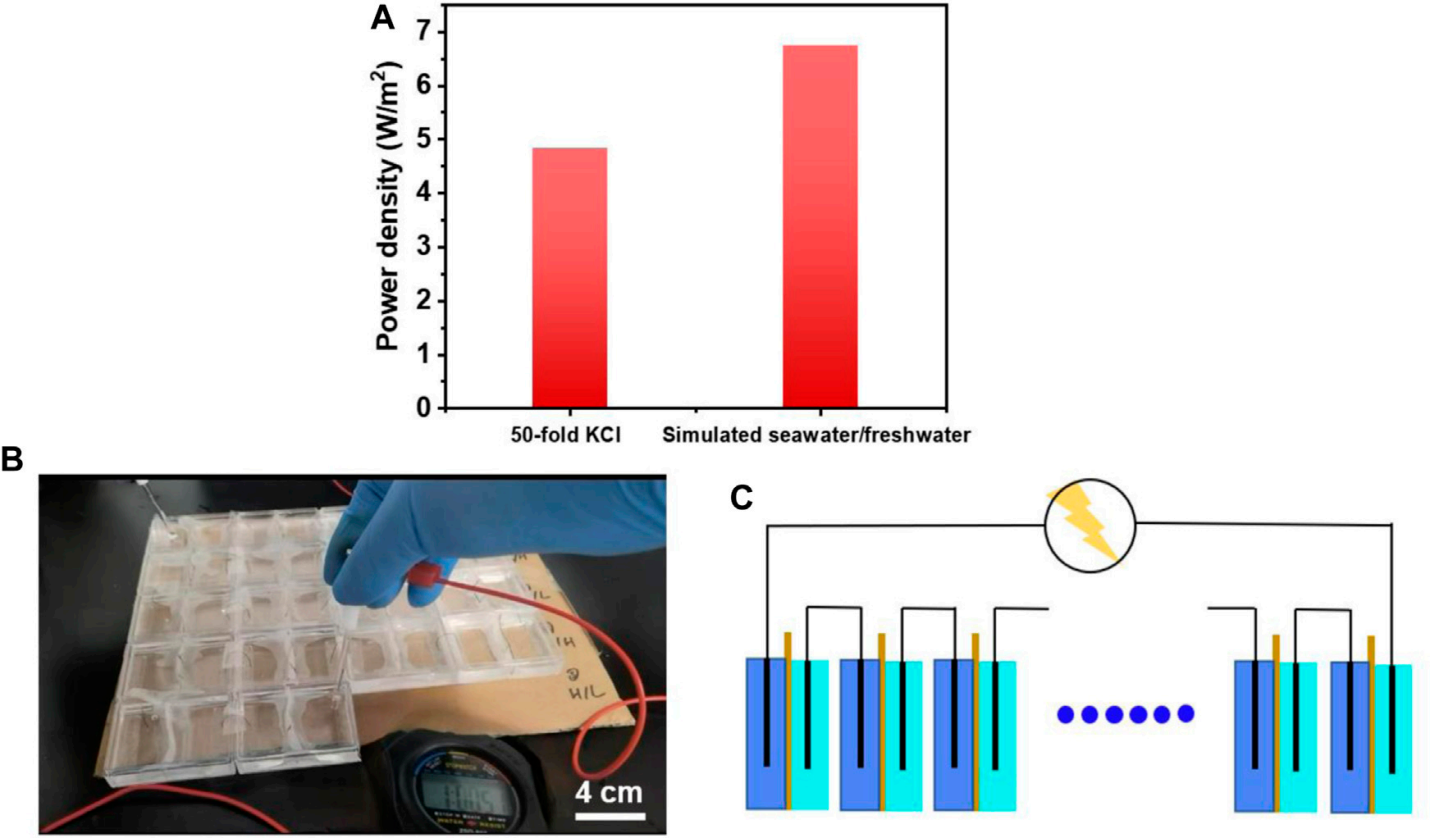
**(A)** Power density of dual network fiber-hydrogel membrane in the 50-fold concentration gradients of KCl and simulated seawater/freshwater; **(B)** The experiment of lightening the stopwatch with 18 homemade RED devices; **(C)** The RED devices were connected in series to provide the facilities with electricity.

To further evaluate the osmotic energy harvesting of dual-network membrane, the simulated seawater/river water containing several kinds of ions was obtained and the power density was higher than that in the condition of 50-fold KCl (6.75 W/m^2^), (Figure 6A). What’s more, to verify the actual effect of membrane, RED devices were made and connected with homemade Ag/AgCl electrodes in series. After adding high and low concentration solution, we observed that the stopwatch was lightened (Figure 6B). This phenomenon proved the application potential of the dual-network fiber-hydrogel membrane. It was noted that the voltage of multiple connected RED devices met the demand of electronic facilities (Figure 6C) and made them work.

## 4 Conclusion

In summary, the combination of N-filter paper and the same charged hydrogel could form dual-network fiber-hydrogel membrane, which had good ion selectivity, high transference number (>0.9) and energy conversion efficiency (>32.5%) under different concentration gradients for harvesting osmotic energy. The performance of fiber-hydrogel membrane-based osmotic power generator was treated and the power density was 4.84 W/m^2^ under 50-fold KCl and 6.75 W/m^2^ under the condition of simulated seawater/river water, respectively. Furthermore, through more experiments and ion transport mechanism analysis, these results formed because of the synergies of the surface charge from N-filter paper fibers and the space charge from hydrogel, which was different from other results. Meanwhile, it demonstrated the opportunities and potential to supply electric power for electronic devices.

## Data Availability

The original contributions presented in the study are included in the article/Supplementary Material, further inquiries can be directed to the corresponding author.
