# Supplementary material for: Dual-network fiber-hydrogel membrane for osmotic energy harvesting
Source: Front Chem. 2024 May 9;12:1401854. doi: 10.3389/fchem.2024.1401854 (PMC11112087; doi:10.3389/fchem.2024.1401854)
Supplement: Supplementary file 1 [file DataSheet1.pdf]

Supporting Information

Dual-Network Fiber-Hydrogel Membrane for Osmotic Energy Harvesting

*Licheng Cao, Huiqing Wu

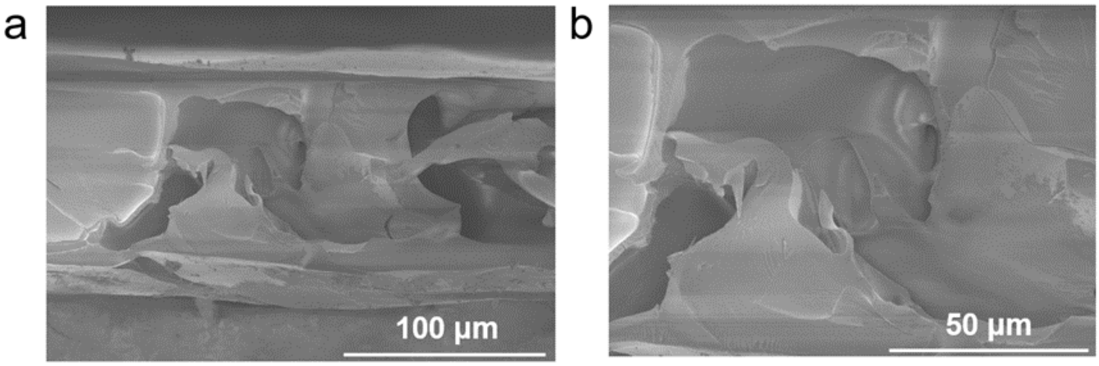

Figure S1. (a-b)The cross-sectional SEM images of pure charged hydrogel.

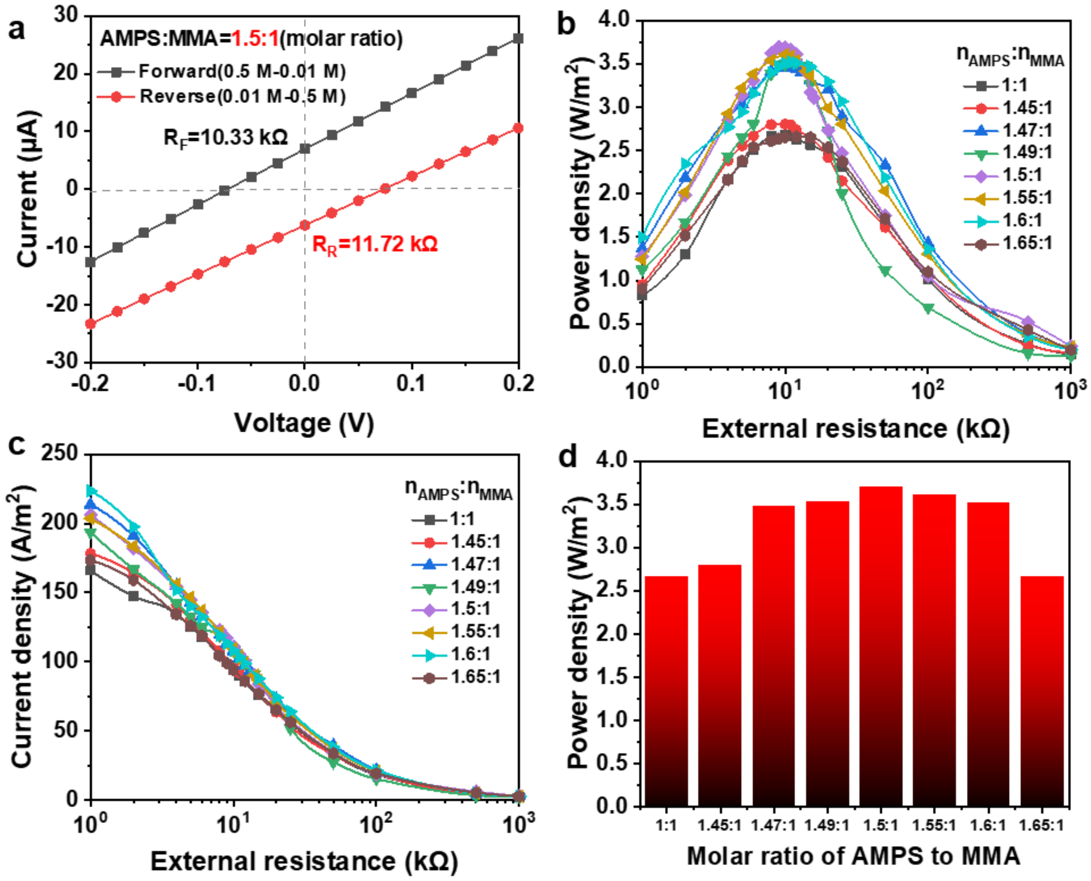

Figure S2. The osmotic energy conversion of N-filter paper fiber-hydrogel membrane with different molar ratio of AMPS and MMA. (a) The I-V curves of membranes at 50-fold concentration gradient with different gradient directions when molar ratio was 1.5:1. The curves of (b) power density and (c) current density at different external resistance with different molar ratio; (d) The column chart of power density with different molar ratio.

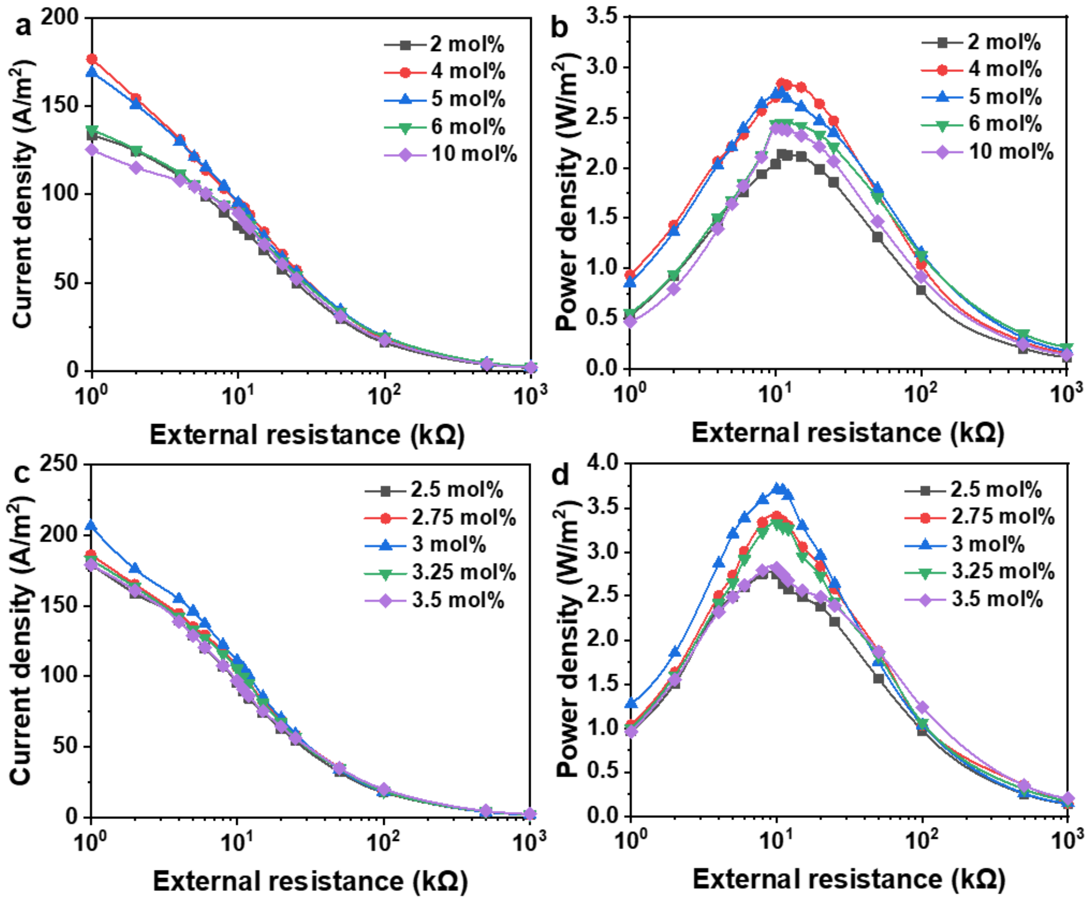

Figure S3.The curves of (a, c) current density and (b, d) power density at different external resistance with different crosslinking agent molar fraction when the molar ratio of AMPS to MMA was 1.5:1.

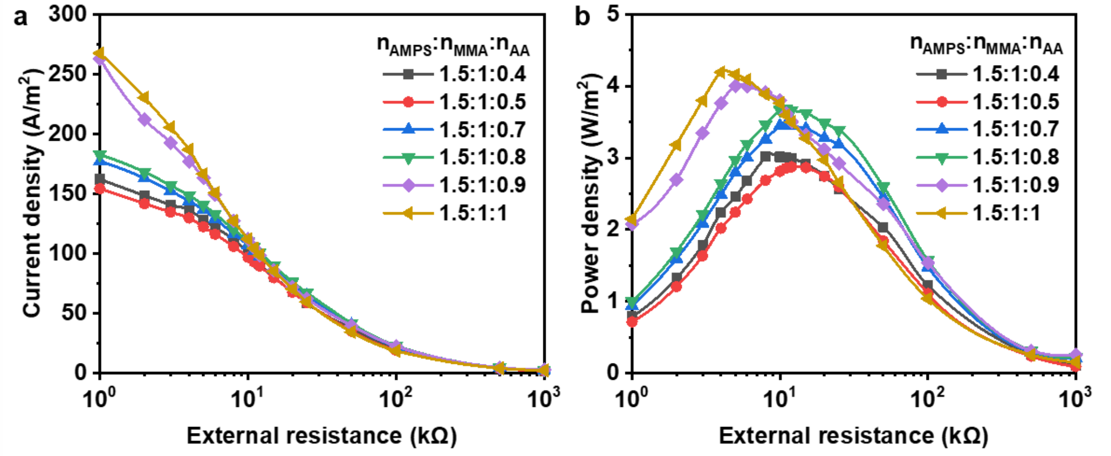

Figure S4.The curves of (a) current density and (b) power density at different external resistance with different molar ratio of AMPS, MMA and AA. (The crosslinking agent was 3 mol%, relative to the total monomers.)

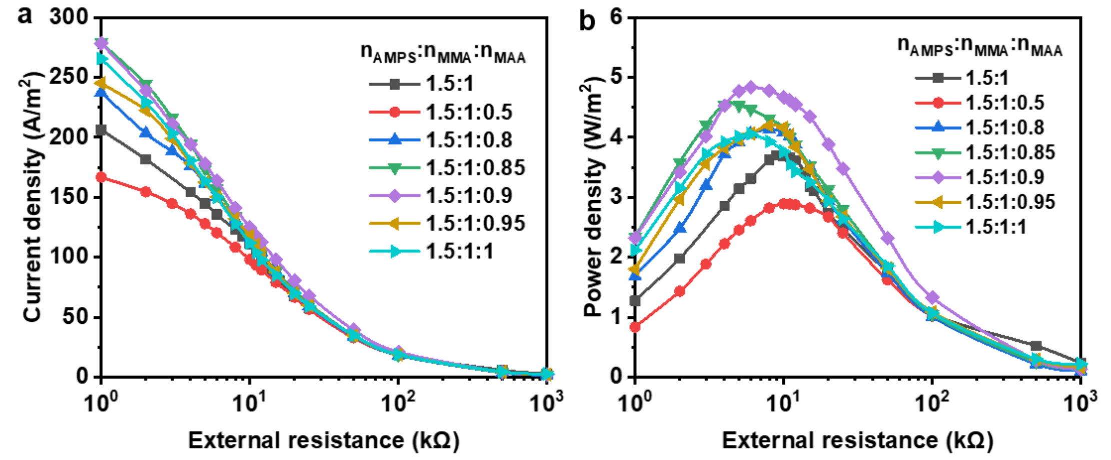

Figure S5.The curves of (a) current density and (b) power density at different external resistance with different molar ratio of AMPS, MMA and MAA. (The crosslinking agent was 3 mol%, relative to the total monomers.)

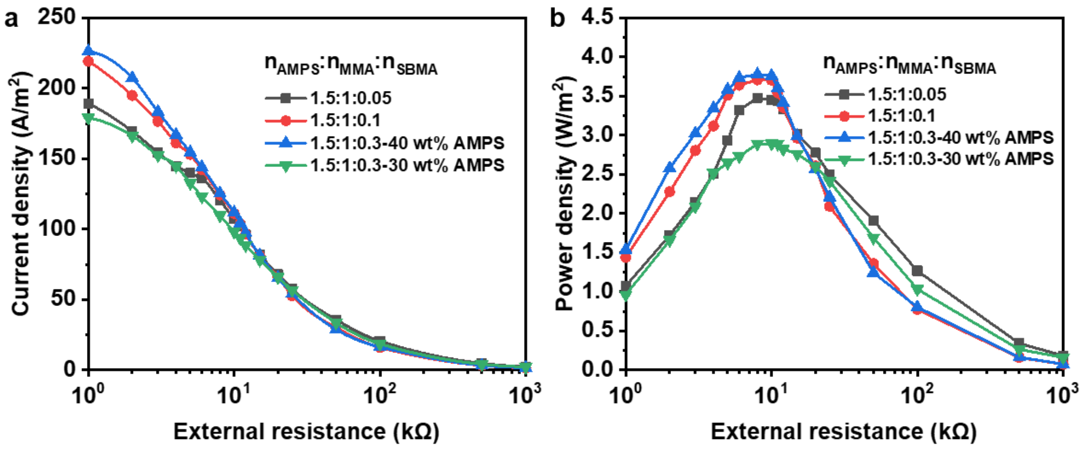

Figure S6.The curves of (a) current density and (b) power density at different external resistance with different molar ratio of AMPS, MMA and SBMA. (The crosslinking agent was 3 mol%, relative to the total monomers.)

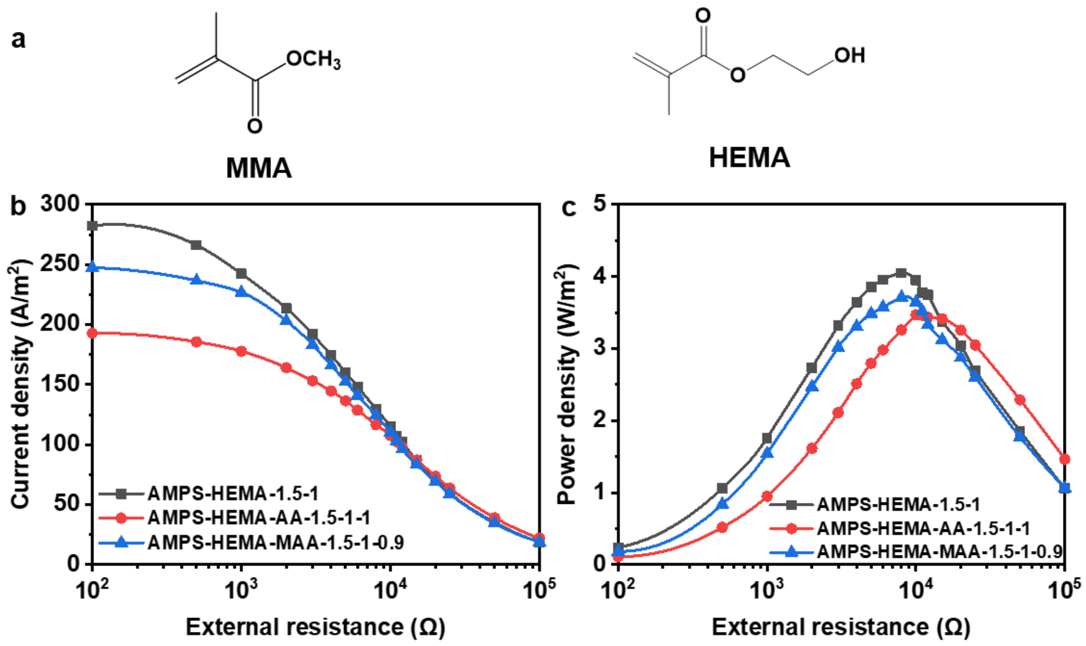

Figure S7. (a) The chemical structure of MMA and HEMA. The curves of (b) current density and (c) power density at different external resistance with acertainmolar ratio of AMPS, HEMA and AA (MAA). (The crosslinking agent was 3 mol%, relative to the total monomers.)

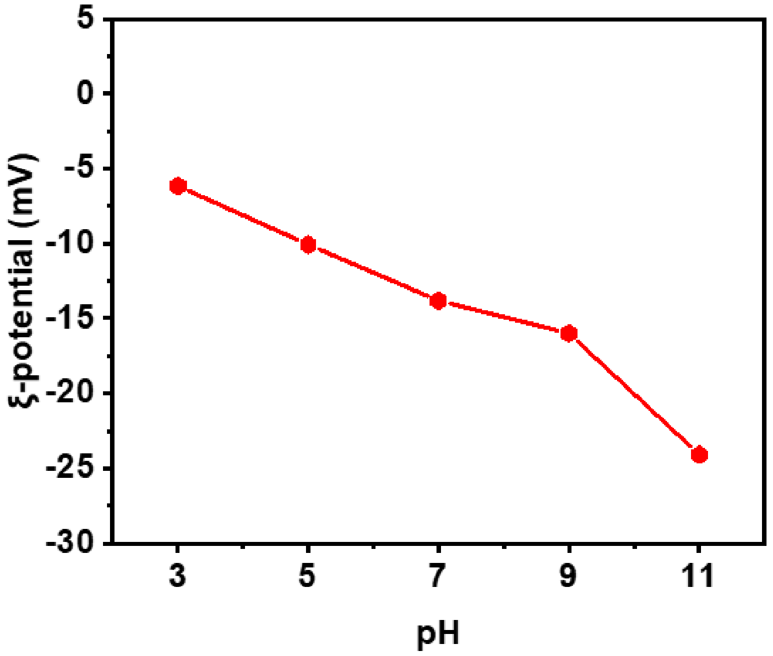

Figure S8. Zeta potential of the pure hydrogel when pH ranged from 3 to 11.
